# The serum level of a novel lipogenic protein Spot 14 was reduced in metabolic syndrome

**DOI:** 10.1371/journal.pone.0212341

**Published:** 2019-02-14

**Authors:** Yen-Ting Chen, Ping-Huei Tseng, Fen-Yu Tseng, Yu-Chiao Chi, Der-Sheng Han, Wei-Shiung Yang

**Affiliations:** 1 Graduate Institute of Clinical Medicine, College of Medicine, National Taiwan University, Taipei, Taiwan; 2 Division of Gastroenterology, Department of Internal Medicine, National Taiwan University Hospital, Taipei, Taiwan; 3 Division of Endocrinology & Metabolism, Department of Internal Medicine, National Taiwan University Hospital, Taipei, Taiwan; 4 Department of Physical Medicine and Rehabilitation, National Taiwan University Hospital Bei-Hu Branch, Taipei, Taiwan; 5 Center for Obesity, Lifestyle and Metabolic Surgery, National Taiwan University Hospital, Taipei, Taiwan; 6 Graduate Institute of Medical Genomics & Proteomics, College of Medicine, National Taiwan University, Taipei, Taiwan; 7 Hepatitis Research Center, National Taiwan University Hospital, Taipei, Taiwan; Mahidol University, THAILAND

## Abstract

Spot 14 (S14) protein is primarily expressed in adipogenic tissues. Compared to wild type, S14 knockout mice had better resistance to diet-induced obesity and glucose tolerance. However, the association between serum S14 level and metabolic variables in humans has never been investigated. The objectives of this study were to evaluate the associations between serum S14 concentrations with components of metabolic syndrome (MetS). A total of 327 subjects were recruited in this cross-sectional study and categorized by presence of MetS. The mean serum levels of S14 were significantly lower in subjects with MetS than those without (87.1±26.3 μg/L vs. 107.3±40.2 μg/L, *p*<0.001). In addition, the subjects with central obesity, low high density lipoprotein-C (HDL-C) or hypertriglyceridemia also had significantly lower S14 levels in comparison to those without. Adjusted with age and sex, diagnosis of MetS (β = -0.227, *p*<0.001), central obesity (β = -0.176, *p* = 0.001), low HDL-C (β = -0.149, *p* = 0.005), and high triglyceride (TG) (β = -0.198, *p*<0.001) were negatively associated with log transformation of serum S14 levels (logS14). With 25% logS14 increased, the risk of MetS (OR 0.65, 95% CI, 0.51–0.82, *p*<0.001), central obesity (OR 0.72, 95% CI, 0.58–0.89, *p* = 0.002), low HDL-C (OR 0.76, 95% CI, 0.61–0.95, *p* = 0.015) or high TG (OR 0.65, 95% CI, 0.51–0.83, *p* = 0.001) was reduced with a dose response trend. Our analysis revealed that patients with MetS had lower serum S14 levels than those without. Negative associations existed between MetS, central obesity, high TG, low HDL-C and logS14.

## Introduction

The prevalence rate of metabolic syndrome (MetS) in the modern society has increased tremendously, probably due to poor dieting and exercise habits [1]. According to the current definition, the MetS is a cluster of several metabolic abnormalities [2]. MetS increases the risk for many cardiovascular diseases, such as ischemic heart disease and stroke [2]. Obesity is the major contributing factor for the MetS [3]. Both hyperplasia (increased cell number) and hypertrophy (increased cell size) of adipocytes can lead to obesity [4–6]. Studying adipocyte biology may help us identify potential solutions to fix the problems of obesity and the MetS. In the past decades, adipose tissue has been considered as a genuine endocrine tissue [7]. Many adipokines, such as leptin and adiponectin have been extensively characterized [8, 9]. Recently, a lipogenic factor S14 (Spot 14, also called thyroid hormone responsive protein, THRSP) has been identified [10]. S14 is the 14^th^ spot of proteins which were previously annotated on the two-dimensional gel for their differential expression in the liver of hypothyroid rats treated with or without thyroid hormone [10]. The *S14* gene is mainly expressed in the lipid-producing tissues, such as liver, adipose tissue and lactating mammary glands [11]. Its expression in hepatocytes can be rapidly and tremendously induced by triiodotyronime (T3), glucose and insulin [12, 13]. Transfection of S14 antisense oligonucleotides into cultured hepatocytes repressed the expression or the activity of certain lipogenesis-related enzymes, such as ATP-citrate lyase, fatty acid synthase, and malic enzyme [14]. Moreover, the S14-knockout mice were shown to be resistant to diet-induced obesity accompanied with better insulin sensitivity and glucose tolerance [15]. These knockout animals had reduced amount of adipose tissues, but normal amount of lean mass [15].

The information regarding the function of S14 in human subjects is limited. The basal S14 mRNA levels in the subcutaneous adipose tissues of obese subjects were shown to be lower than normal controls [16, 17]. The S14 mRNA levels in the adipose tissues of non-obese individuals, but not of the obese subjects, were dramatically suppressed after fasting for 48 hours [16]. These observations suggest that the expression of S14 in the adipose tissues of the obese subjects is dysregulated [16]. However, the association between serum S14 level and metabolic variables in humans has not been investigated. The aim of this study is to evaluate the relationship of serum S14 concentrations with anthropometric and metabolic factors in human subjects.

## Material and methods

### Human subjects

A total of 541 ostensibly healthy individuals were recruited at the Health Management Center of National Taiwan University Hospital (NTUH). Written informed consent was obtained from each subject. In accordance with the Declaration of Helsinki and approved guidelines, the Research Ethics Committee of NTUH approved the study including the protocol, informed consent form, and applicable recruiting materials (NTUH, No. 201204030RIB). Physical examination and blood chemistry were performed. To avoid the possible effects of inflammation and/or abnormal thyroid status, people who were diagnosed as Hepatitis B, Hepatitis C, rheumatoid arthritis, systemic lupus erythematosus, hyperthyroidism or hypothyroidism were excluded. Subjects who were taking medicine for common cold, steroid, non-steroid anti-inflammatory drugs, and thyroid therapy drugs were also excluded. At the end, 327 individuals were included for the study.

### Data collection, measurement, and determination

The basic characteristics including age, gender, body height (BH), body weight (BW), body waist and blood pressure (BP) on the right arm were collected. Body mass index (BMI) was calculated as BW (in kilograms) divided by BH (in meters) squared. The body fat percentage (BFP) was determined by the body composition analyzer DX-300 (Jawon Medical, Gyeongsan, Korea).

Blood samples were drawn from antecubital vein after overnight fast for at least 8 hours. Postprandial blood glucose level was examined 2 hours after taking a standardized meal (750 kcal, including 54% carbohydrates, 30% protein, and 16% fat) prepared by the nutrition room of NTUH [18]. Biochemical assays including alanine transaminase (ALT), aspartate transaminase (AST), fasting plasma glucose (FPG), postprandial plasma glucose (PPG), hemoglobin (Hb), glycated hemoglobin (HbA1c), free thyroxine (fT4), thyroid-stimulating hormone (TSH), high-density lipoprotein cholesterol (HDL-C), low-density lipoprotein cholesterol (LDL-C), total cholesterol (T-C), triglyceride (TG), creatinine (Cre), and uric acid (UA) were performed following the manufacturers’ instructions of the routine tests by the Department of Laboratory Medicine, National Taiwan University Hospital as previously described [18–20].

### Definition of metabolic syndrome and related components

Subjects with MetS were defined by the modified National Cholesterol Education Program, Adult Treatment Panel III (NCEP ATP III) criteria for Asians [21]. The five MetS characteristic components includes: (ⅰ) Central obesity: waist circumference ≧90 cm in men and ≧80 cm in women; (ⅱ) high blood pressure: blood pressure ≧130/85 mmHg or known treatment for hypertension; (ⅲ) high fasting glucose: fasting plasma glucose level ≧100 mg/dl (5.6 mmol/l) or known treatment for diabetes; (ⅳ) low HDL-C: serum HDL-C level <40 mg/dl (1.03 mmol/l) in men and <50 mg/dl (1.29 mmol/l) in women; and (ⅴ) high TG: serum triglyceride level ≧150 mg/dl (1.7 mmol/l) [22, 23]. MetS was diagnosed with at least three of the above five criteria. The cut-off points of BFP for men were 25.0% and for women were 35.0%.

### Human S14 immunoassay

A competitive ELISA for human serum S14 was developed as previously described [19]. Briefly, 100 μl of human recombinant S14 proteins (100 μg/L, diluted in cold 1x PBS; cat no. ag3721, ProteinTech, Chicago, IL) were coated on the Polystyrene MaxiSorp 96-well plates (Nunc A/S, Roskilde, Denmark) and incubated at 4°C overnight. The next day, the plates were blocked with 150 μl/well blocking buffer [PBS-0.05% Tween 20 (PBS-T) with 1% BSA], and incubated on the orbital shaker at room temperature (RT) for 90 minutes. The solutions were then discarded and 100 μl/well serum samples or standards were added and incubated on a rotor at RT. After one hour, rabbit anti-S14 polyclonal antibody (catalog no. 13054-1-AP, ProteinTech, Chicago, IL) were added (50 μl per well) and shaken for 2 hours at RT. After washing with PBS-T three times, horseradish peroxidase-conjugated goat anti-rabbit IgG polyclonal antibody (GTX213110-01, Irvine, CA, USA) was added (100 μl per well) and incubated for 1 hour at RT. Following five times of washing with PBS-T, the solutions were then discarded and the plates were pad-dried on a paper towel. Then 100 μl 3, 3’, 5, 5’-tetramethylbenzidine (TMB) solution (KPL, Gaithersburg, MD) were added to each well for coloration for 15 minutes. The reaction was stopped by adding 100 μl 2.0 M H_2_SO4 to each well. The absorbance was measured immediately at 450 nm by microplate reader (VERSA max, Munich, Germany). The standard curve was obtained using four-parameter logistic model. The minimum detection limit of the assay was 7.7 μg/L. The coefficients of variation (CV) of intra-assay and inter-assay were 10.5% and 6.1% respectively.

### Statistical analysis

Numerical data were expressed as mean ± standard deviation (SD). Differences of numerical data between groups were analyzed by two-tailed *t* test. Differences of categorical data between groups were analyzed by Chi-square test. The skewness and kurtosis of the data was checked with normality test. If the distribution of the variable was not in normal distribution, the data of the variable were log transformed before subsequent analysis. The associations between demographic, anthropometric, or laboratory parameters with serum levels of S14 were further analyzed by linear regression after log transformation. To estimate the odds ratios (OR) of metabolic syndrome and its different components in relation to log-transformed S14, we divided the log-transformed S14 into quartiles. Logistic regression models were used to examine the OR with 95% confidence intervals (CI). P values <0.05 were considered as statically significant. All statistical tests were performed with IBM SPSS version 22.0 statistical package (IBM Corporation, Armonk, USA).

## Results

Ninety-eight subjects of a total of 327 subjects (30.0%) were diagnosed with MetS. The mean age of them was 53.2 years old. The mean age of the subjects with MetS or without MetS (non-MetS) was not different (Table 1). In all subjects, 61.5% were men. There were more male subjects in the MetS group than the non-MetS group (75.5% vs. 55.5%, *p*<0.001) (Table 1). In comparison to the non-MetS, the MetS group had higher BH, BW, BMI, BFP, waist, SBP, DBP, ALT, Cre, UA, FPG, PPG, HbA1c, and TG, but lower HDL-C (Table 1). There was no difference of thyroid function tests (fT4 and TSH) between these two groups.

**Table 1:** Demographic, anthropometric, and biochemical characteristics of the subjects with metabolic syndrome (MetS) or without metabolic syndrome (Non-MetS).

	MetS (N = 98)	Non-MetS (N = 229)	*p*
Variables			
Age (years)	54.2 ± 8.4	52.8 ± 10.2	0.183
Gender (% male)	75.5%	55.5%	<0.001*
BH (cm)	167.2 ± 8.3	164.1 ± 8.7	0.002*
BW (kg)	76.3 ± 13.4	62.4 ± 10.6	<0.001*
BMI (kg/m^2^)	26.9 ± 3.2	23.1 ± 2.8	<0.001*
BFP (%)	29.3 ± 4.1	25.5 ± 5.3	<0.001*
Waist (cm)	94.6 ± 8.4	83.1 ± 8.1	<0.001*
SBP (mmHg)	129.4 ± 17.0	115.1 ± 12.7	<0.001*
DBP (mmHg)	79.3 ± 11.9	68.7 ± 9.4	<0.001*
AST (U/L)	22.4 ± 7.9	21.5 ± 8.2	0.341
ALT (U/L)	26.1 ± 14.4	20.1 ± 10.7	<0.001*
Cre (μmol/L)	79.6 ± 35.4	70.7 ± 17.7	0.005*
UA (μmol/L)	386.7 ± 95.2	339.1 ± 83.3	<0.001*
FPG (mmol/l)	6.1 ± 2.0	5.2 ± 0.8	<0.001*
PPG (mmol/l)	7.9 ± 3.5	6.5 ± 2.2	<0.001*
HbA1c (%)	6.2 ± 1.3	5.6 ± 0.4	<0.001*
HDL-C (mmol/l)	1.1 ± 0.2	1.4 ± 0.3	<0.001*
LDL-C (mmol/l)	3.2 ± 0.8	3.1 ± 0.8	0.533
T-C (mmol/l)	5.1 ± 1.0	5.1 ± 0.9	0.669
TG (mmol/l)	2.3 ± 1.6	1.1 ± 0.6	<0.001*
fT4 (pmol/L)	12.9 ± 2.6	14.2 ± 3.9	0.308
TSH (μIU/ml)	2.4 ± 1.3	2.2 ± 1.4	0.384

*: *p* < 0.05

Numerical variables are presented at mean ± standard deviation. BW: body weight, BMI: body mass index, BFP: body fat percentage, SBP: systolic blood pressure, DBP: diastolic blood pressure, AST: aspartate transaminase, ALT: alanine transaminase, Cre: creatinine, UA: uric acid, FPG: fasting plasma glucose, PPG: postprandial glucose, HbA1c: glycated hemoglobin, HDL-C: high density lipoprotein cholesterol, LDL-C: low density lipoprotein cholesterol, T-C: total cholesterol, TG: triglyceride, fT4: free thyroxine, TSH: thyroid-stimulating hormone, MetS: metabolic syndrome, Non-MetS: without metabolic syndrome

**Fig 1:**
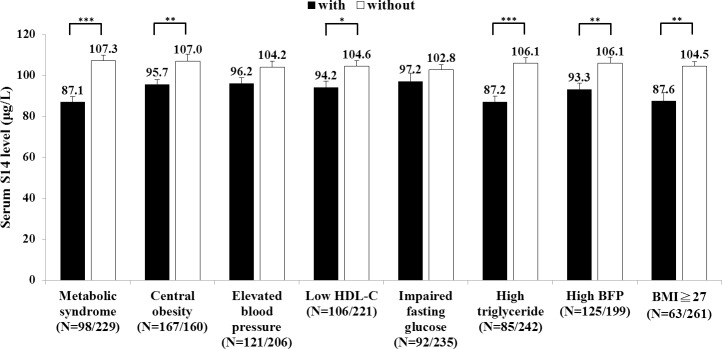
Comparison of serum S14 levels between the subjects with and without MetS or related components. The S14 levels of the subjects with and without the components were respectively shown with black and white bars. The cut-off points of body fat percentage (BFP) for men were 25.0% and for women were 35.0%. The data were shown as mean and standard error. N shows the individual number. * *p*<0.05, ** *p*<0.01, *** *p*<0.001.

The mean serum S14 level of the subjects with MetS was significantly lower than that of the non-MetS subjects (87.1±26.3 μg/L vs. 107.3±40.2 μg/L, *p*<0.001) (Fig 1). Further examination of each component of the MetS showed that the subjects with central obesity (95.7±30.4 μg/L vs. 107.0±43.4 μg/L, *p* = 0.006) and high TG (87.2±26.3 μg/L vs. 106.1±39.8 μg/L, *p*<0.001) had significantly lower serum S14 levels than these without (Fig 1). In addition, the subjects with low HDL-C also had significantly lower S14 levels than those without (94.2±31.3 μg/L vs. 104.6±40.0 μg/L, *p* = 0.02) (Fig 1). There was no significant differences in S14 levels between the subjects with and without elevated blood pressure or with and without impaired fasting glucose (Fig 1). Interestingly, the subjects with high body fat (93.3±33.9 μg/L vs. 106.1±39.1 μg/L, *p* = 0.003) or BMI (87.6±31.8 μg/L vs. 104.5±38.3 μg/L, *p* = 0.001) also had significantly lower S14 levels than those without (Fig 1). We also noted that the female subjects had significantly higher serum S14 levels than the males (112.7±42.9 μg/L vs. 94±32.1 μg/L, *p*<0.001).

Univariate linear regression analysis revealed a negative association between age and logS14 (β = -0.004, *p* = 0.029). Female gender was significantly associated with higher logS14 (β = 0.177, *p*<0.001). Adjusted with age and gender, logS14 was significantly related to the MetS in a negative manner (Table 2). Using individual component of the MetS as the independent variable, the logS14 was in negative association respectively with central obesity, low HDL-C, and high TG (Table 2). In contrast, the relationship between the logS14 and elevated blood pressure or impaired fasting glucose was not significant (Table 2). In multivariate linear regression with age, gender and all five components of the MetS as independent variables in the model, age (β = -0.120, *p* = 0.030), gender (β = -0.237, *p*<0.001), central obesity (β = -0.139, *p* = 0.016) and high TG (β = -0.152, *p* = 0.011) were significantly associated with the logS14 levels.

**Table 2:** Linear regression analysis with components of metabolic syndrome as independent variables and log transformation of serum S14 level (logS14) as dependent variable, adjusted with age and sex.

	logS14		
Independent variable	ß	95% C.I.	*p*
Diagnosis of MetS	-0.227	-0.239, -0.088	<0.001*
Central obesity	-0.176	-0.185, -0.048	0.001*
Elevated blood pressure	-0.028	-0.094, 0.056	0.616
Low HDL-C	-0.149	-0.179, -0.032	0.005*
Impaired fasting glucose	-0.035	-0.106, 0.055	0.533
High triglyceride	-0.198	-0.230, -0.069	<0.001*

*: *p*<0.05

ß: parameter estimate; 95% C.I.: 95% confidence interval. MetS: metabolic syndrome. HDL-C: high density lipoprotein cholesterol

In logistic regression models, we divided logS14 into quartiles and estimated the risk for the MetS and its five components with the adjustment of age and gender. The increment in logS14 level reduced risk for MetS, central obesity, low HDL-C, or high TG with a dose response trend (Table 3). Each quartile increment in logS14 was associated with reduced risk for the MetS (OR 0.65, 95% CI, 0.51–0.82, *p*<0.001), central obesity (OR 0.72, 95% CI, 0.58–0.89, *p* = 0.002), low HDL-C (OR 0.76, 95% CI, 0.61–0.95, *p* = 0.015) and high TG (OR 0.65, 95% CI, 0.51–0.83, *p* = 0.001) respectively by 35%, 28%, 24% and 35%. On the other hand, the risk for elevated blood pressure and impaired fasting glucose was not altered with various logS14 levels (Table 3).

**Table 3:** Odds ratio (95% CI) for MetS and its components according to the quartiles of logS14 with the adjustment of age and gender.

		Odds ratio	95% C.I.
Metabolic syndrome		0.65	0.51, 0.82
	1^st^ quartile	1.00	
	2^nd^ quartile	0.64	0.34, 1.22
	3^rd^ quartile	0.47	0.24, 0.93
	4^th^ quartile	0.25	0.12, 0.54
	*P*_trend_: <0.001*		
Central obesity		0.72	0.58, 0.89
	1^st^ quartile	1.00	
	2^nd^ quartile	0.77	0.41, 1.44
	3^rd^ quartile	0.65	0.34, 1.24
	4^th^ quartile	0.35	0.18, 0.68
	*P*_trend_: 0.002*		
Elevated blood pressure		0.94	0.75, 1.16
	1^st^ quartile	1.00	
	2^nd^ quartile	0.74	0.39, 1.42
	3^rd^ quartile	0.95	0.49, 1.84
	4^th^ quartile	0.74	0.38, 1.47
	*P*_trend_: 0.551		
Low HDL-C		0.76	0.61, 0.95
	1^st^ quartile	1.00	
	2^nd^ quartile	1.01	0.53, 1.91
	3^rd^ quartile	0.82	0.42, 1.58
	4^th^ quartile	0.41	0.20, 0.84
	*P*_trend_: 0.015*		
Impaired fasting glucose		0.96	0.76, 1.22
	1^st^ quartile	1.00	
	2^nd^ quartile	0.76	0.38, 1.52
	3^rd^ quartile	0.68	0.33, 1.40
	4^th^ quartile	0.93	0.45, 1.92
	*P*_trend_: 0.745		
High triglyceride		0.65	0.51, 0.83
	1^st^ quartile	1.00	
	2^nd^ quartile	0.85	0.44, 1.65
	3^rd^ quartile	0.55	0.27, 1.14
	4^th^ quartile	0.24	0.10, 0.57
	*P*_trend_: 0.001*		

*: *p* < 0.05

HDL-C: high density lipoprotein cholesterol, MetS: metabolic syndrome

## Discussion

The present study is the first to evaluate the association of serum S14 levels with the MetS and related components. Increased serum level of S14 was found to associate with decreased risk of the MetS, central obesity, high TG and low HDL-C. Aside from liver and adipose tissue, the mRNA expression level of S14 had been examined in non-lipogenic tissues such as brain, pituitary, lung, heart, kidney, spleen, and testis [11, 24]. However, the S14 mRNA was barely detectable in those tissues by Northern blots [11, 24]. Since the relative S14 mRNA expression levels in fat depots was reported to be 10 fold higher than that in liver in animals [11], it is plausible that the serum S14 may mainly come from the adipose tissue. Previous study in humans showed that S14 mRNA expression in the adipose tissue of obese subjects was lower than non-obese controls [17]. Consistent with this, our study also revealed that obese subjects had lower serum S14 levels than non-obese individuals. As previous studies have shown that S14 facilitates fatty acid synthesis [14], it is reasonable that S14 may have more direct associations with lipid-related components of MetS (central obesity, high TG and low HDL-C) rather than glucose or blood pressure parameters. Some studies have reported that S14 proteins localized in nucleus of hepatocytes and pre-adipocytes [17, 25, 26]. It was suggested that S14 might be an important transcription factor or co-activator for regulating the expression of several lipogenic genes [25, 27]. In mammary gland, S14 is important for epithelial proliferation and milk fat production [28]. Kinlaw and his colleagues reported that S14 protein was overexpressed in most lipogenic breast cancers [29, 30], and promoted breast cancer cell growth and survival. S14 overexpression in lipogenic human breast cancer cells, such as T47D, SKBR3 and MCF7, accelerated cell growth but not in mammary epithelial MCF10a cell; whereas knockdown with short inhibitory RNA (siRNA) the cell growth were inhibited [29]. Therefore, S14 may also function as an important modulator of tumorigenesis in human breast.

The reduced serum S14 level in human MetS is reminiscent of the case of adiponectin [9, 31]. Adiponectin is a protein hormone which is involved in a number of metabolic processes, including regulating glucose levels as well as fatty acid oxidation [31]. It is secreted from adipose tissue and is very abundant in plasma [31]. The levels of adiponectin are lower in obese subjects than in lean subjects [32]. Weight reduction accompanied by improved insulin sensitivity increases the plasma levels of adiponectin [9]. Similar to adiponectin, the blood S14 level is reduced in obesity and the MetS in our study. Consistently, the basal S14 mRNA levels in the subcutaneous adipose tissues of obese subjects were reported to be lower than normal controls [16, 17]. The cause of reduced expression of S14 both at the mRNA and protein levels in the MetS and obesity is still not clear. We speculate that certain inhibitory factors related to insulin resistance may suppress the expression of S14 similar to the case of adiponectin [9, 31]. However, the exact mechanism remains to be explored.

In this study, we found that female gender had higher serum S14 levels. The percentages of MetS were also higher in males than in females (36.8% vs. 19.0%). Previous nation-wide epidemiological survey in Taiwan showed that the age-standardized prevalence of MetS was 14.3% (16.1% in men and 13.3% in women) [33]. They also found that the prevalence of MetS increased with age and this increase was most obvious in postmenopausal women [33]. Therefore, a higher percentage of MetS in males than in females in our subjects could be a selection bias. However, gender effect was adjusted in further analyses, and the association of S14 with the MetS remained unchanged. The cause of this gender difference of S14 expression in humans is unclear. The major source of S14 is adipose tissue [11]. The fat distributions vary in adult men and women [34]. The sex hormones such as progesterone, estrogens, and androgens have also been reported to be involved in the regulation of adipose tissue metabolism [25, 34, 35]. Progesterone administration significantly increased the expression level of S14 mRNA in subcutaneous white adipose tissue (WAT) of female rats but had no effect in inguinal, epididymal and retroperitoneal WAT of male rats [34]. Chicken *THRSP* gene was also shown to be regulated by estrogen directly through an estrogen receptor binding site located in the *THRSP* promoter [25]. In addition, compared to intact male chickens, THRSP gene expression was upregulated by 2.2-fold in the livers of capons, whose testes had been surgically incised then the levels of androgen were significantly reduced [35]. These regulations of S14 expression by sex hormones in adipose tissue and liver may contribute to the observed gender difference of S14 levels in humans in our study. Whether this is also true for humans awaits further examination.

Our analysis revealed a negative association of age with logS14. So far, there is no human study discussing the mRNA or protein expression levels of S14 with age. A previous study of rats showed that with the age increasing from one to ten months, the mRNA expression levels of S14 increased about 4.48 fold in the liver, but reduced nearly 50% in the epididymal fat [11]. The mechanism of this difference is still unclear, and whether this is also true in humans remains unknown.

There were several limitations in this report. First, the antibody used in our immunoassay was polyclonal. Existence of a paralogous protein (S14R) which has 32% homology with S14 [36] may cause some cross reaction in our immunoassays. However, the expression level of S14R was demonstrated to be far lower than S14, approximately 1/10 in the liver and 1/100 in the adipose tissues at the mRNA level [37]. The use of our S14 immunoassay is reliable and has been reported to be related human thyroid function before [19]. Second, this is a cross-sectional study. The causal relationship between serum S14 levels and the metabolic abnormalities cannot be inferred, although we speculate that reduced S14 expression is the result of the metabolic abnormality. Third, we did not have sufficient biochemical data such as insulin levels to directly address the relationship between S14 and insulin-resistance in this study.

## Conclusion

We found that serum S14 had negative association with MetS, central obesity, high TG, low HDL-C in humans. Females tend to have higher serum S14 levels than males. Blood S14 declines with age.
